# The role of HCV proteins on treatment outcomes

**DOI:** 10.1186/s12985-015-0450-x

**Published:** 2015-12-15

**Authors:** Kattareeya Kumthip, Niwat Maneekarn

**Affiliations:** Department of Microbiology, Faculty of Medicine, Chiang Mai University, Chiang Mai, 50200 Thailand

**Keywords:** Antiviral agent, Hepatitis C virus, Interferon, Mutation, Ribavirin

## Abstract

For many years, the standard of treatment for hepatitis C virus (HCV) infection was a combination of pegylated interferon alpha (Peg-IFN-α) and ribavirin for 24–48 weeks. This treatment regimen results in a sustained virologic response (SVR) rate in about 50 % of cases. The failure of IFN-α-based therapy to eliminate HCV is a result of multiple factors including a suboptimal treatment regimen, severity of HCV-related diseases, host factors and viral factors. In recent years, advances in HCV cell culture have contributed to a better understanding of the viral life cycle, which has led to the development of a number of direct-acting antiviral agents (DAAs) that target specific key components of viral replication, such as HCV NS3/4A, HCV NS5A, and HCV NS5B proteins. To date, several new drugs have been approved for the treatment of HCV infection. Application of DAAs with IFN-based or IFN-free regimens has increased the SVR rate up to >90 % and has allowed treatment duration to be shortened to 12–24 weeks. The impact of HCV proteins in response to IFN-based and IFN-free therapies has been described in many reports. This review summarizes and updates knowledge on molecular mechanisms of HCV proteins involved in anti-IFN activity as well as examining amino acid variations and mutations in several regions of HCV proteins associated with the response to IFN-based therapy and pattern of resistance associated amino acid variants (RAV) to antiviral agents.

## Background

**Fig. 1:**
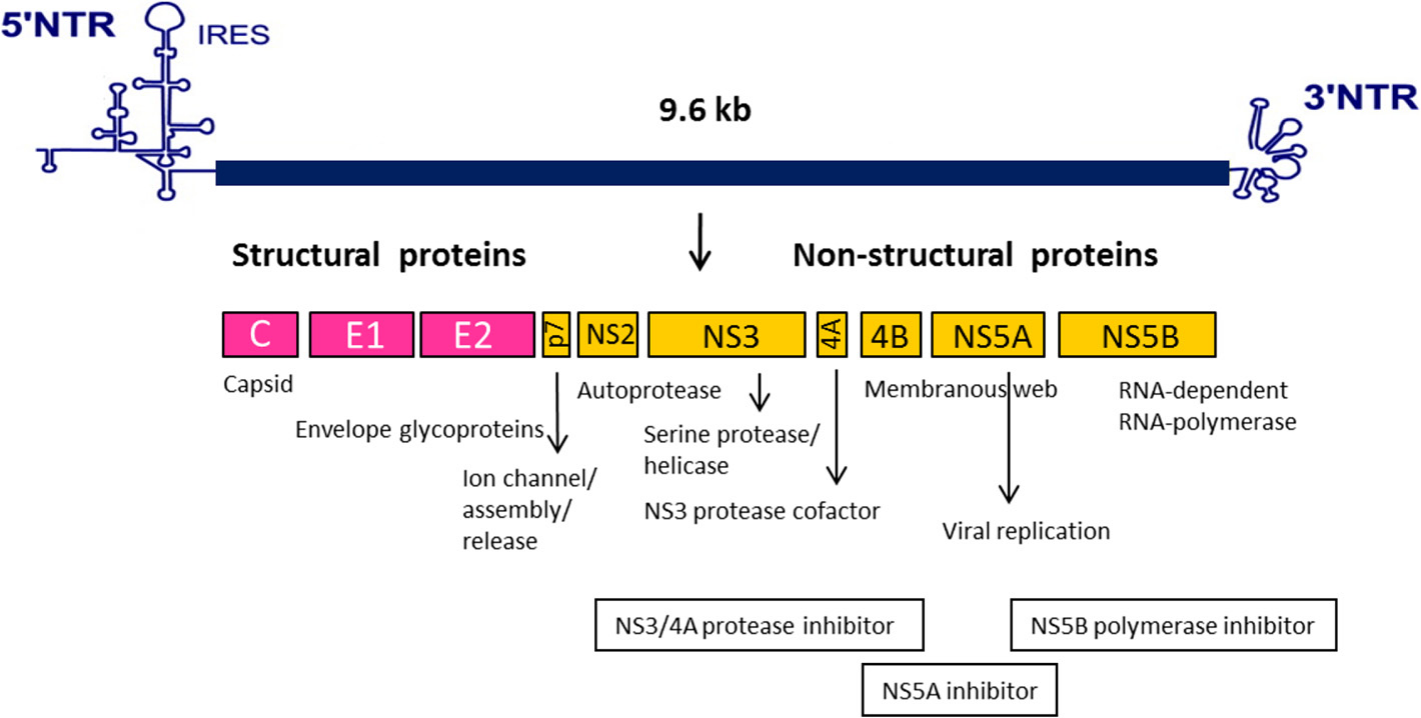
HCV genomic organization, HCV proteins and targets for direct-acting antiviral agents. The HCV genome, having approximately 9.6 kilobases (kb), contains a 5'-nontranslated region (NTR), an internal ribosome entry site (IRES), a long open reading frame encoding polyprotein precursor of about 3,000 amino acid residues and a 3'-NTR. The polyprotein precursor is processed by both host and viral enzymes to release functional structural and non-structural proteins. HCV NS3/4A, HCV NS5A, and HCV NS5B are targets for antiviral agents [1, 2]

Hepatitis C virus (HCV) belongs to the *Hepacivirus* genus which is part of the *Flaviviridae* family. HCV is a small enveloped virus with a positive single-stranded RNA genome containing approximately 9,600 nucleotides which encodes for a large polyprotein of about 3,000 amino acids. The polyprotein precursor is cleaved by the host and viral proteases into three structural proteins (core, E1, E2) and seven nonstructural proteins (p7, NS2, NS3, NS4A, NS4B, NS5A, NS5B) [1, 2] (Fig. 1). HCV core protein is a highly conserved RNA-binding protein, which presumably forms the viral nucleocapsid and plays a role in pathogenesis [3–5]. E1 and E2 envelope glycoproteins are essential components of the HCV virion and necessary for viral entry and fusion [6, 7]. P7 could act as a calcium ion channel and has an important role in viral maturation and release [8, 9]. NS2 is a transmembrane protein required for NS2/3 autoprotease activity that cleaves the site between the NS2 and NS3 junction [10–12] while NS3 is the protease and NTPase/helicase [13, 14]. NS4A serves as a cofactor of the NS3 protease activity and NS4B functions as a membrane anchor for the replication complex [15, 16]. NS5A is a hydrophilic phosphoprotein needed for viral replication [17, 18]. NS5B, a RNA-dependent RNA polymerase (RdRp), is a key enzyme for viral replication promoting synthesis of new RNA genomes [19, 20]. With the lack of a proof-reading activity and error correction mechanisms of the viral RdRp, the high genetic variability and high degree of mutation rate of the HCV are occurred, allowing for rapid adaptation and lead to a genetically variant pool of viruses within the infected individual [21, 22]. Due to the diversity of the genome, HCV is classified into 7 major genotypes and 67 subtypes [23]. HCV genotypes are distributed in different parts of the world. Genotypes 1–3 are widely distributed throughout the world, genotypes 1 and 2 are endemic in West Africa while genotype 3 is endemic in India. Genotypes 4 and 5 are prevalent in Africa and genotype 6 in Southeast Asia. The distribution of genotype 7 has not been fully evaluated [24, 25].

Life cycle of HCV includes entry into the host cell, uncoating of the viral genome, translation of viral proteins, polyprotein processing, viral genome replication, assembly and release of virions. All these events occur outside the nucleus of the host cell [26]. Following initial binding of the HCV particle to the host cellular receptors, scavenger receptor class B member 1 (SRB1) [27] and CD81 [28] via the E2 glycoprotein, the particle engages in further interactions with several entry factors including tight junction proteins claudin 1 (CLDN1) [29], occludin (OCLN) [30], epidermal growth factor receptor (EGFR), the ephrin receptor [31], and finally enters cells by receptor-mediated endocytosis. The viral RNA genome is released into the cytoplasm and subsequently translated at the rough endoplasmic reticulum (ER). After translation, the viral proteins, in conjunction with host cell factors, induce the formation of a membranous web where the RNA genome replication occurs [26]. The positive sense RNA genome is generated through a negative strand intermediate and is packaged into the viral particle containing the core and envelope proteins which accumulates to the ER and lipid droplets [32]. HCV virions are presumably formed by budding through the ER, or an ER derived compartment, and are exited the cell via the secretory pathway [33].

HCV infection occurs globally and remains a serious health problem worldwide. Nowadays, about 150 million people, representing 2–3 % of the world’s population, are chronically infected with HCV and more than 350,000 people die from HCV-related liver diseases each year [34]. The severity of HCV infection ranges from a mild illness to a serious sickness that leads to chronic hepatitis, liver cirrhosis and hepatocellular carcinoma (HCC). Only a minority of HCV-infected patients can clear the virus spontaneously during acute infection [35]. Innate immune responses are the first line of defense against viral infections and interferons (IFNs) are important cytokine responsible for the induction of an antiviral state of the cells for elimination of HCV during acute infection [36]. However, the majority of patients are unable to clear the virus. Approximately 50–80 % of acute HCV infections persist and progress into chronic infection and approximately 4–20 % of patients with chronic hepatitis C develop liver cirrhosis within 10–20 years. Annually, about 1–5 % of patients with cirrhosis are at risk of developing HCC” [35]. The viral persistence is related to both host and viral factors. The virus has developed several strategies to escape host innate immune responses by different mechanisms [37]. The mechanisms that protect HCV from IFN-mediated innate immune reactions are not completely understood, but involve interruption of IFN induction pathway, IFN-stimulated genes (ISGs) production, or direct antagonism of effector systems by viral proteins [37, 38]. A protective vaccine for HCV infection is not available yet and although therapeutic options are improving they are still limited as well as some patients still resist to the treatment. Development and improvement of antiviral therapies and effective vaccines are still needed.

## Treatment of HCV infection and rate of response

For many years, the combination of IFN-α and ribavirin was the approved treatment regimen for chronic HCV infection. IFNs are a family of cytokines released by host cells in response to various stimuli including virus infection. IFNs induce expression of multiple antiviral effector proteins working against virus replication [36]. IFN-α therapy leads to a rapid decline in HCV RNA levels in serum [39, 40]. Ribavirin is a nucleoside analogue and possesses activity against several RNA and DNA viruses, however, the exact mechanism of action of ribavirin against HCV is still unknown [41]. The combination of IFN-α and ribavirin therapy led to great improvements in SVR rates [42–44].

Further improvement was achieved by the development of pegylated interferon alpha (Peg-IFN-α), in which a large molecule of polyethylene glycol is covalently attached to recombinant IFN-α resulting in an active molecule with a longer half-life, better pharmacokinetic profile, and better rate of virologic response [45, 46]. The commercially available forms of IFN-α used for hepatitis C (α2a, α2b and consensus IFN) have somewhat different potencies in vitro but appear to yield similar response rates in treated patients [47, 48]. For patients who are chronically infected with HCV, the former standard of care recommendation was to use Peg-IFN-α plus ribavirin for 48 weeks for patients infected with genotype 1 or 24 weeks for patients infected with genotype 2 or 3 and for genotypes 4, 5 and 6, it has been suggested that patients were treated in a similar manner as those for patients with genotype 1 [46]. This treatment regimen led to SVR rates ranging from 42 to 46 % in patients infected with genotype 1, 76–80 % in patients infected with genotype 2 or 3, and 50–77 % in patients infected with genotypes 4, 5, and 6 [48–50]. Overall sustained responses occur in about one-half of patients but the likelihood of response varies greatly, depending on viral and host characteristics [51, 52]. The key host factors associated with response to IFN include IL28B genotype, race, and fibrotic stage [53, 54] while viral load and viral genotype are important viral factors associated with SVR [52, 54]. In addition, many problems associated with IFN-based regimens such as considerable side-effects, expense, and length of treatment lead to many patients not being able to tolerate the full course of treatment, leading to treatment failure [55].

Since 2011, a number of direct acting antiviral agents (DAAs), which specifically target hepatitis C viral proteins including NS3/4A, NS5A and NS5B, have been used to improve the treatment of HCV infection. First generation NS3/4A protease inhibitors, including telaprevir and boceprevir, were approved by the United States Food and Drug Administration (FDA) for HCV genotype 1 infection in 2011 [56–59]. Both compounds were approved as triple combinations with Peg-IFN-α and ribavirin, and this regimen increased SVR rates in HCV genotype 1 infected patients to 66–75 % among patients who had not previously received treatment for their infection (treatment-naïve) and 59–64 % in patients who had not responded to previous treatment (treatment-experienced) [56–60]. A second-generation NS3/4A protease inhibitor, simeprevir [61], was approved in 2013. By using simeprevir in combination with Peg-IFN-α and ribavirin, the rates of SVR in treatment-naïve HCV genotype 1 infected patients were 75–85 % [62–64].

Several NS5A inhibitors have been reported such as ledipasvir (formerly GS-5885) [65], ombitasvir, formerly known as ABT-267 [66], and daclatasvir (BMS-790052) [67]. Ledipasvir and daclatasvir were approved in 2014 for use in combination with sofosbuvir (NS5B inhibitor). Triple therapy with Peg-IFN-α, ribavirin, and the NS5A inhibitor daclatasvir led to SVR in 59–100 % of treatment-naïve HCV genotype 1 and 4 infected patients, according to drug dosage and HCV genotype [68].

There are 2 types of NS5B inhibitors, nucleoside/nucleotide analogs and non-nucleotide analogs. Nucleotide analogs such as mericitabine [69] and sofosbuvir [70] act as false substrates for the HCV-RdRp and this leads to chain termination after being incorporated into the newly synthesized viral RNA [71, 72], and non-nucleotide analogs, such as dasabuvir [73] bind to several discrete sites on the NS5B polymerase, which leads to conformational protein changes. Sofosbuvir was approved in 2013 as part of a combination treatment with IFN and ribavirin and demonstrated extraordinary efficacy for treatment-naïve patients infected with HCV genotype 1 and 4 with 90 % of SVR rate [74–76]. The use of triple IFN-containing regimens with the DAAs is associated with side effects that can be severe. The IFN-free regimens have been further considered by using a combination of several DAAs with or without ribavirin. For example, the combination of sofosbuvir plus ribavirin in genotype 2 infected patients for 12 weeks showed an SVR rate of about 95–97 % [77].

In October 2014, the FDA approved Harvoni as the first DAA combination drug that does not require administration in combination with interferon or ribavirin. This daily, single tablet drug consists of the NS5A inhibitor (ledipasvir) and the NS5B polymerase inhibitor (sofosbuvir). In phase III clinical trials which enrolled 1,518 participants including treatment-naïve, treatment-experienced and patients with cirrhosis, it was shown that these groups of patients achieved an SVR rate of between 96 and 99 % within 12–14 weeks of the administration of the drug [78, 79]. This new IFN-free therapy allows for a simplified and shortened therapy duration to 12–24 weeks for all HCV genotypes.

## HCV proteins which mediate IFN resistance

**Fig. 2:**
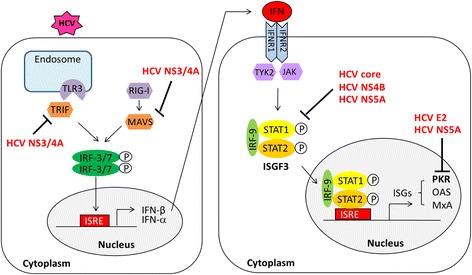
Classical pathways of type I IFN induction and HCV mediating IFN resistance. HCV dsRNA is detected by the retinoic acid inducible gene I (RIG-I) and the toll-like receptor 3 (TLR3) and subsequently triggers the cascade of adaptor proteins, mitochondrial antiviral signaling protein (MAVS) and TIR-domain containing adapter-inducing interferon-β (TRIF), respectively. This leads to the activation of the type I IFN induction pathway. Binding of IFNs to their cellular receptors activates an intracellular signaling cascade via the Jak/STAT signaling pathway and leads to the up-regulation of a number of interferon-stimulated genes (ISGs) expression [80–82]

IFNs are cytokines which are part of the host’s natural immune response to the presence of pathogens, such as viruses, bacteria, parasites or tumor cells [36]. After the binding of IFNs to their specific receptors on the target cell surface, an intracellular signaling cascade, the Janus kinase (Jak) and signal transducer and activator of transcription (STAT) pathway is activated. This leads to the up-regulation of a number of IFN-stimulated genes (ISGs) and expression of multiple antiviral effector proteins to eradicate the virus [80–82] (Fig. 2). IFN-α was first shown to have beneficial effects in patients with chronic HCV infection in 1986 [40] and has been used for treatment of HCV infection until now. The failure of IFN-α-based treatment to eradicate HCV infection is influenced by multiple factors [51, 54]. It is believed that host factors and viral factors, particularly viral load and HCV genotype are important factors contributing to the difference in response to the IFN therapy [83]. HCV genotype 1 infected patients achieve poorly sustained rates of response (42–52 %) to IFN-α-based therapy compared to those infected with HCV genotypes 2 and 3 (78–86 %) [52]. So far, it has been suggested that several HCV proteins are responsible for the inhibition of the antiviral effects of IFN-α [84]. The expression of the whole HCV polyprotein as well as single HCV proteins of core, E2, NS3/4A, NS4B or NS5A have been shown to antagonize the antiviral effect mediated by IFN-α in many different ways as summarized in Fig. 2.

### HCV core

The core protein of HCV interferes with the IFN signaling pathway. It has been shown that the HCV core induced the expression of the suppressor of the cytokine signaling protein 3 (SOCS-3) and SOCS-1 in cultured cells, which in turn antagonized IFN-α action by blocking the Jak/STAT pathway and the expression of ISGs [85, 86]. The effect of core protein on the down-regulated expression of IFN-induced antiviral genes was described [87]. Additionally, the inhibitory effects of the HCV core protein on IFN-induced phosphorylation and nuclear translocation of STAT1 in cell cultures were demonstrated. The HCV core protein binds directly to STAT1, suggesting a model by which the binding of HCV core to STAT1 resulted in the decrease of STAT1 phosphorylation and disruption of ISGs transcription [88, 89].

### HCV E2

The E2 protein of HCV was reported to be involved in mediating IFN-α resistance through the inhibition of protein kinase R (PKR) [90]. HCV E2 contains a peptide of eight amino acids that are identical to a sequence in PKR, four of which act as autophosphorylation sites in PKR. Next to this region, E2 contains another consecutive stretch of four amino acids identical to the phosphorylation site of the eukaryotic initiation factor 2 alpha subunit (eIF2α) [82]. This region is known as the PKR-eIF2α phosphorylation homology domain (PePHD). Binding of the PePHD derived from HCV genotype 1 to the cellular PKR abolishes its kinase activity and blocks its inhibitory effect on protein synthesis in vitro [90]. This may lead to viral protein translation taking place during virus infection and IFN treatment. However, these effects were not detectable for PePHD sequences derived from HCV genotypes 2 and 3. It has been hypothesized that the interaction of the PePHD with the PKR may result in a relatively enhanced resistance of HCV-1 isolates to IFN-α-based antiviral therapy [90].

### HCV NS3/4A

The HCV protease disrupts the innate immune response and IFN induction pathway. IFNs are induced in the presence of the HCV viral genome sensing by pattern-recognition receptors, retinoic acid inducible gene I (RIG-I) and toll-like receptor proteins (such as TLR3) [37, 91]. It was shown that HCV NS3/4A protease cleaves host proteins including the mitochondrial antiviral signaling protein (MAVS) [92, 93], and the TIR-domain containing adapter-inducing interferon-β (TRIF) [94], adaptor proteins of RIG-I and TLR3 signaling pathways, respectively. This leads to inhibition of the innate immune response and the IFN induction pathway. The HCV NS3/4A protein has also been shown to block the phosphorylation and nuclear translocation of the interferon regulatory factor 3 (IRF-3), a key component of the downstream signal of both RIG-I and TLR3 resulting in a significant reduction of the transcription of IFN-α-inducible genes [95, 96].

### HCV NS4B

It has been shown that the expression of HCV NS4B in cell culture inhibited the protection of the cells by IFN-α treatment from vesicular stomatitis virus infection. The NS4B protein of HCV reduced the IFN-α-induced phosphorylation level of STAT1 and the expression levels of type I interferon receptors and a reporter driven by the IFN-stimulated response element (ISRE) promoter [97].

### HCV NS5A

The NS5A protein of HCV binds to and inactivates PKR in vitro. This binding is dependent on the PKR binding domain (PKRBD, codons 2209–2274) [98, 99]. This interaction presumes to allow viral protein synthesis to occur during IFN treatment. A number of studies have demonstrated the inhibitory effects of HCV NS5A protein on the IFN-induced Jak/STAT signaling pathway [100–103]. It has been shown that HCV NS5A blocked the ISRE promoter activity and IFN-induced STAT1 phosphorylation and its nuclear translocation resulted in inhibition of the IFN-induced ISGs expression [100, 101, 103]. It has been proposed that the interaction between the C-terminal region of NS5A and STAT1 is responsible for the anti-IFN activity of NS5A [102, 103].

## Amino acid variations associated with the response to IFN-based therapy

In addition to the study of how HCV proteins counteract IFN activity, cloning and sequencing approaches for HCV isolates derived from patient sera with known virologic responses to antiviral therapy have been investigated. The clinical importance of amino acid variability within several functional regions of HCV proteins such as core, E1, E2, NS3, NS4B, NS5A and NS5B in correlation with the responses to IFN therapy have been described [104–143].

### HCV core

The significance of amino acid mutations in the core protein of HCV and the response to IFN-based therapy has been described well in genotype 1b. Previous studies have demonstrated that substitutions of amino acid residues 70 (R70Q) and/or 91 (L91M) in the core region were significant factors independently associated with a non-virological response (NVR) to IFN and ribavirin combination therapy [104, 105]. Substitutions in this region were also used for predicting the response to IFN in Japanese patients with HCV genotype 1b [106]. Moreover, the HCV core mutants R70 and L91 were shown to be resistant to IFN in vitro. Theses mutants were associated with a decrease in IFN-induced phosphorylation of STAT1 and STAT2, and the expression of ISGs through enhancement of the expression of SOCS-3 and interleukin 6 [107, 108].

### HCV E1/E2

Several studies have demonstrated that mutations within the PePHD of the E2 protein of HCV genotypes 1b and 3a showed no correlation with the clinical outcomes in patients treated with IFN-α [109–112]. However, it has been demonstrated that PePHD of genotypes 2a and 2b had multiple amino acid variations and one particular motif, “RGQQ-” at the N-terminus of PePHD showed a close relationship with IFN resistance [113]. Another study showed that the presence of substitutions in a N-terminal variable region (codons 617–641) in the C-terminal region of E2 showed a significant correlation with a low viral load and a sustained response to IFN treatment [114]. Furthermore, a study into recombinant 1a and 3a HCV genotypes identified that amino acid substitutions at positions 345 and 348 of E1 and 414 of E2 increased IFN-α resistance [115]. It was shown that amino acid changes I348T in E1 protein of HCV genotype 1a (H77), F345V in E1 and V414A in E2 of genotype 3a (S52) increased viral fitness and that I348T and F345V/V414A mutants enhanced viral entry and release, respectively [115].

### HCV NS3

In the HCV replicon system, several mutations within the NS3 gene that led to the enhancement of replication efficiency have been reported. The HCV isolates from patients harboring amino acid substitutions in the NS3 protein (R1283G, P1112R, and S1496M) showed a slower decrease of HCV RNA than those without mutations at those positions during IFN-α-based therapy [116]. Additionally, it has been reported that the HCV 1b-infected patients with a better anti-IFN response (>3.5 log decline of viral RNA by day 28) have higher number of amino acid substitutions in the NS3 protein than those of the patients with a poor response (<1.4 log decline of viral RNA by day 28) [117]. Recently, it has been demonstrated that increases in amino acid variations in the NS3 protein of HCV genotypes 1a, 1b, 3a, and 3b are associated with the response to Peg-IFN and ribavirin combination therapy [118]. Amino acid variations within the full-length NS3, particularly in protease and helicase domains of NS3 of HCV genotype 1a, as well as the full-length NS3/helicase domain of HCV genotype 1b from responding patients treated with Peg-IFN and ribavirin are significantly more frequent than those from treatment failure groups [118]. In addition, use of an HCV replicon cell line which was exposed to IFN-α and IFN-β revealed that substitutions S1269Y, K1270R, and R1135K in NS3 protein were associated with IFN resistance [119]. The possible reasons that may explain why these mutations may lead to IFN resistance based on the fact that NS3 is known as a bifunctional enzyme with serine protease activity on the N-terminal domain and RNA helicase on its C-terminal domain [120]. The NS3 helicase is essential for HCV RNA replication and also plays a role in viral particle assembly [121]. Conceivably, amino acid substitutions in this region or particular position may affect the function of NS3 and contribute to the acquisition of IFN resistance. Correspondingly, Numba et al. [119] also described these amino acid substitutions of S1269Y, K1270R, and R1135K in the NS3 may contribute to the acquisition of IFN resistance. To clarify these points, further analysis, such as the characterization of HCV replicon cells re-established by the transfection of these HCV replicon RNAs into Huh7 cells, will be necessary [119].

### HCV NS4B

Genetic analysis of HCV replicon cells resistance to IFN-α and IFN-β exposure revealed that they all share a single common amino acid substitution in the NS4B (Q1737H) suggesting that these genetic alterations are involved in their IFN-resistant phenotype [119]. In addition, a rapid initial HCV RNA decline of ≥1.5 log 10 IU/mL at week 2 of IFN-based therapy is associated with a higher frequency of non-conservative amino acid exchanges within the complete NS4B protein when compared to patients with a non-rapid HCV RNA decline [122].

### HCV NS5A

**Fig. 3:**
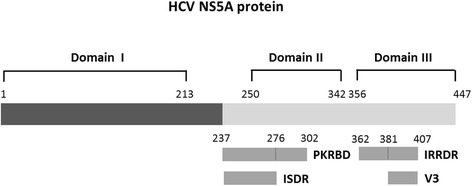
HCV NS5A protein. Several regions within the NS5A proteins of HCV play a role in the IFN sensitivity of HCV including the interferon-sensitivity-determining region (ISDR; codons 2209–2248 of HCV genome corresponding to amino acid residues 237–276 of NS5A protein), the interferon and ribavirin resistance determining region (IRRDR; codons 2334–2379 or amino acid residues 362–407 of NS5A), the protein kinase R binding domain (PKRBD; codons 2209–2274 or amino acid residues 237–302 of NS5A), and the variable region 3 (V3; codons 2353–2379 or amino acid residues 381–407 of NS5A) [17, 98, 123, 135, 137]

The initial data which supported a significant role of amino acid variations within the NS5A protein of HCV and the response to IFN therapy was first described by Enomoto et al. in [123]. A correlation of a high number of mutations in a IFN-α sensitivity determining region (ISDR; codons 2209–2248) (Fig. 3) comprising 40 amino acids within the C-terminal part of the NS5A protein and SVR to IFN-α monotherapy in HCV genotype 1b infected patients was described in Japan [124]. The viruses obtained from patients who achieved an SVR showed at least four mutations within the ISDR. Subsequently, the correlation of ISDR mutations with the response to IFN treatment was investigated by different groups in Japan, Europe and the United States. Many of the studies were from Japan and were able to confirm the strong correlation between ISDR mutations and treatment response [114, 125–128]. However, contradictory data have been reported from other parts of the world, particularly from Europe and the United States. The correlation of ISDR mutations with IFN-αsensitivity was not able to be verified [111, 129–132]. The discrepancy may be explained, at least in part, by host and viral factors. It is well documented that host factors, particularly the race of the subjects, are important factors contributing to HCV treatment responsiveness [133]. In addition to ISDR, the composition and number of mutations found in the interferon and ribavirin resistance determining region (IRRDR, residues 2334–2379, genotype 1) have been shown to be associated with response to treatment [107, 134, 135]. A local accumulation of mutations around the region so-called V3 (variable region 3; codons 2356–2379) within the C-terminal part of the NS5A was reported to correlate with the response to Peg-IFN/ribavirin therapy in HCV genotype 1 isolates [134, 136–138]. Furthermore, genetic analysis of HCV replicon cells which were resistant to IFN treatment revealed that particular substitutions in the NS5A protein (M2174V, T2319A/N, T2242N, F2256L) are associated with the resistance to IFN activity [119]. It has been thought that NS5A blocks IFNs by interacting with PKR, a double-strand RNA-dependent protein kinase. Amino acid substitutions in the NS5A protein may exert the function of PKR. The possibility is also considered that some cellular factors, either alone or in combination with viral factors, contributed to the acquisition of IFN resistance [119].

### HCV NS5B

The NS5B protein of HCV has been one of a number of putative targets of nucleoside analogs, including ribavirin [139]. It has been suggested that the HCV NS5B variant F415Y conferred resistance to ribavirin in HCV genotype 1a infected patients [140]. Substitutions of E124K and I85V identified in the NS5B protein are found to be closely associated with early viral clearance at 8 weeks in patients who were treated with IFN and ribavirin combination therapy and were infected with the HCV genotype 1b and had a high level of viremia [141]. Moreover, particular amino acid changes in the NS5B protein of HCV have been shown to correlate with the outcomes of combined-IFN plus ribavirin therapy. Sequence analysis of NS5B revealed that mutations at amino acid residues 309, 333, 338 and 355 of NS5B occurred more frequently in a group of patients exhibiting an SVR or an end-of-treatment response than in a group of patients showing a negative-response [142]. Furthermore, it has been shown that mutations in NS5B, particularly in the thumb domain and at amino acid position 389, correlate with the viral load and response to IFN therapy of HCV genotype 1b [143].

## HCV mutations associated with the response to antiviral agents

To date, several DAAs targeting the key components of virus replication have been approved for the treatment of HCV infection. Advances in using these drugs with or without a combination of Peg-IFN/ribavirin have improved SVR rates. However, resistance to antiviral agents of HCV isolates have been documented. The patterns of resistant variants or specific mutations are influenced by multiple parameters including the specific inhibitor, viral genotypes/subtypes and level of drug selective pressure [144, 145]. In addition, some resistant mutations exist as natural polymorphisms in each genotype/subtype. The pattern of specific mutations resistant to each antiviral agent are shown in Table 1.

**Table 1:** Amino acid substitutions associated with the resistance to different direct-acting antiviral agents

HCV protein targets	DAAs	Pattern of mutations
NS3/4A	Boceprevir	V36M/A^a^, T54A/V/S^a^, V55A^a^, R155K/T^a^, A156T/S/V^a^, V158I, D168N, V/I170A/T/L, L/M175L
	Telapevir	V36G/L/M/A^a^, T54A/V/S^a^, S122A/G/R, R155K/T^a^, A156T/S/V^a^, D168A/H/T/V
	Simeprevir	V36M, F43S**,** **Q80K** ^a^, S122A/R, R155T/K^a^, A156T/V, **D168Q**/A/H/T/V/E^a^, V/I170A/T/L
NS5A	Daclatasvir	M28V/A/T, Q30R/E/H, L31F/V/M^a^, Q54H/N/Y, H58D, Q62R/E, A92K/T, Y93H/N/C^a^
	Ledipasvir	M28T, Q30R/H, L31V, **Y93H**/C
NS5B	Sofosbuvir	**S282T**, I434M, T179A, M289L, I293L, M434T, H479P, L159F/L320F

^a^Amino acid substitution represents the significant mutations that are clearly associated with reduced the response to treatment

*DAAs* direct-acting antiviral agents. Bolded amino acid substitutions indicate mutations frequently found to confer resistance to DAAs. Q80K for genotype 1a; D168Q for genotype 3; Y93H for genotype 1b; S282T for genotypes 1a, 1b, and 2a. Adapted from reviews [144, 145, 148, 149, 157]

### Resistance to NS3/4A protease inhibitors

The FDA has approved three protease inhibitors, including boceprevir, telaprevir and simeprevir for HCV genotype 1 treatment, still, these are not approved to be used in monotherapy. A number of pre-treatment resistance associated amino acid variants (RAVs) and polymorphisms have been shown to be associated with lower responses to the treatment [146]. Both in vitro and in vivo studies showed that several mutations in the NS3 at positions 36, 155, 156, and 168 conferred broad cross-resistance affecting all of these protease inhibitors [146–148]. Boceprevir and telaprevir showed broad cross-resistance. The RAVs most frequently associated with boceprevir monotherapy are V36M, T54A/S, V55A, R155K, A156S/T/V and V170A. Similar variants are observed for telaprevir except for V55A and V170A [144, 147, 149]. Moreover, the resistance mutation profile is also influenced by HCV genotypes/subtypes. For example, patients infected with HCV genotype 1a showed main selective amino acid mutations at positions 36 and 155 whereas patients with HCV genotype 1b exhibited selective mutations at positions 54, 55, 156 and 170 [144, 145, 150–152]. Substitution of D168Q is generally found in HCV genotype 3 and this natural occurring mutation confers resistance to most protease inhibitors [146, 147]. Simeprevir exhibits high antiviral activity against HCV genotypes 2, 4, 5 and 6 whereas no effect has been observed in genotype 3 [153]. The Q80K mutation is a natural polymorphism detected at baseline in the NS3 sequence. This variant is the most prevalent baseline polymorphism observed in 19–48 % of HCV genotype 1a and appears to be associated with an impaired response to simeprevir [144, 153–155].

### Resistance to NS5A inhibitors

NS5A inhibitors, daclatasvir (BMS-790052) and ledipasvir (GS- 5885), target the binding to domain I of NS5A (amino acid residues 1–213) and this binding leads to blocking of RNA replication and virion assembly [156, 157]. The potent antiviral activity of daclatasvir against HCV replicons from different genotypes has been demonstrated [158, 159]. However, higher rates of virologic responses to daclatasvir have been observed among HCV genotype 1b compared to genotype 1a [160, 161]. The resistance profile of daclatasvir has been shown to be associated with several amino acid changes within domain I of NS5A at positions M28T, Q30R/H, L31V and Y93H for HCV genotype 1a and L31H and Y93H for genotype 1b. These substitutions have been identified in the in vitro replicon system and show a strong correlation with those observed in the clinical outcome [157, 162]. In the case of ledipasvir, baseline pre-treatment mutations found in patients with HCV genotype 1a before being exposed to this drug are M28T, Q30R/H, L31M, Y93C/H. These mutations are associated with reduced susceptibility to treatment response. In the case of genotype 1b, Y93H is the most frequent substitution detected in 100 % of all patients [163].

### Resistance to NS5B polymerase inhibitors

Sofosbuvir was recently approved by the FDA for the treatment of chronic hepatitis C. Substitution of S282T in NS5B has been reported to confer resistance to sofosbuvir in vitro [164, 165]. By using a replicon system to investigate the antiviral activity of sofosbuvir against different HCV genotypes including 1a, 1b, 2a, 2b, and 3a, it was revealed that the S282T mutation is the most common mutation which conferred resistance to sofosbuvir in genotypes 1a, 1b and 2a. For genotype 1a replicons, an additional mutation I434M is observed in combination with S282T. For genotype 2a, at least five additional mutations (T179A, M289L, I293L, M434T, and H479P) have been detected [164, 165]. Recently, mutations of L159F and L320F in the NS5B polymerase that conferred resistance to sofosbuvir have also been reported [166]. Sofosbuvir has a very high barrier to resistance in patients as the resistant mutants are very unfit.

## Conclusions

Treatment of HCV infection has shown a dramatic improvement in recent years since DAAs have been introduced. A combination of antiviral agents with or without IFN/ribavirin improves treatment success and requires shorter treatment durations for patients infected with diverse HCV genotypes. Although IFN-free therapy has increased high SVR, this regimen is generally more expensive than IFN-based regimen. Hence, some groups of patients may require IFN-containing regimen. The results of IFN-based therapy is likely to remain suboptimal due to host factors such as the host immune response, IL28B phenotype, the presence or absence of cirrhosis, and viral factors of viral load or genotypes. Therefore, HCV mediated-IFN resistance may remain an important factor in the failure of treatment in certain parts of the world and needs to be explored. Understanding of viral mutations and their associations with the clinical outcomes of treatment as well as mechanisms of HCV mediated-IFN resistance should contribute to the improvement of therapeutic guidelines and development of high efficacy and tailor made appropriate regimens for chronic hepatitis C patients with different genotypes and special cases.
